# Multi-Features Classification of Prostate Carcinoma Observed in Histological Sections: Analysis of Wavelet-Based Texture and Colour Features

**DOI:** 10.3390/cancers11121937

**Published:** 2019-12-04

**Authors:** Subrata Bhattacharjee, Cho-Hee Kim, Hyeon-Gyun Park, Deekshitha Prakash, Nuwan Madusanka, Nam-Hoon Cho, Heung-Kook Choi

**Affiliations:** 1 Department of Computer Engineering, u-AHRC, Inje University, Gimhae 50834, Korea; subrata_bhattacharjee@outlook.com (S.B.); gusrbs82@gmail.com (H.-G.P.); deekshithadp96@gmail.com (D.P.); nuwanmadusanka@hotmail.com (N.M.); 2 Department of Digital Anti-Aging Healthcare, Inje University, Gimhae 50834, Korea; chgmlrla0917@naver.com; 3 Department of Pathology, Yonsei University Hospital, Seoul 03722, Korea; cho1988@yumc.yonsei.ac.kr

**Keywords:** microscopic biopsy image, wavelet transform, colour features, texture features, multilayer perceptron, neural network, prostate carcinoma, histological sections

## Abstract

Microscopic biopsy images are coloured in nature because pathologists use the haematoxylin and eosin chemical colour dyes for biopsy examinations. In this study, biopsy images are used for histological grading and the analysis of benign and malignant prostate tissues. The following PCa grades are analysed in the present study: benign, grade 3, grade 4, and grade 5. Biopsy imaging has become increasingly important for the clinical assessment of PCa. In order to analyse and classify the histological grades of prostate carcinomas, pixel-based colour moment descriptor (PCMD) and gray-level co-occurrence matrix (GLCM) methods were used to extract the most significant features for multilayer perceptron (MLP) neural network classification. Haar wavelet transformation was carried out to extract GLCM texture features, and colour features were extracted from RGB (red/green/blue) colour images of prostate tissues. The MANOVA statistical test was performed to select significant features based on *F*-values and *P*-values using the R programming language. We obtained an average highest accuracy of 92.7% using level-1 wavelet texture and colour features. The MLP classifier performed well, and our study shows promising results based on multi-feature classification of histological sections of prostate carcinomas.

## 1. Introduction

The diagnosis of medical images is relatively challenging, and the purpose of digital medical image analysis is to extract meaningful information to support disease diagnosis. At present, an enormous quantity of medical images is produced worldwide in hospitals, and the database of these images is expected to increase exponentially in the future. The analysis of medical images is the science of solving medical problems based on different imaging modalities, such as magnetic resonance imaging (MRI), computed tomography (CT), ultrasound, mammography, radiography X-ray, biopsy, and combined modalities, among others. To study detailed tissue structures, pathologists use different histological methods, such as the paraffin technique, frozen sections, and semi-thin sections. The biopsy technique is used to remove a piece of tissue from the body so that it can be analysed in a laboratory. Most cells contained in the tissue are colourless, and therefore this histological section has to be stained to make the cells visible. The most commonly used stain is haematoxylin and eosin (H&E). H&E are chemical compounds used in histology to stain tissue and cell sections pink and blue, respectively [1].

Immunocytochemistry is a common laboratory technique that is used to automatically visualise the localisation of a specific protein and analyse the cells by use of a specific primary antibody that binds it. Paraffin-embedded cell blocks are not commonly used in most laboratories. Preferably, non-paraffin-embedded cells are employed in subcellular localisation studies, and fluorescence is used instead of chromogen. For the determination of protein expression levels, immunofluorescent staining is the commonly used method in research employing cell cultures. Moreover, immunofluorescent staining slides require protection from bright light while preserving cells morphological information. Cytoskeleton-associated protein 2 (CKAP2) and Ki-67 are proteins in humans that are encoded by the *CKAP2* and *MKI67* genes, respectively. Ki-67 is a cellular marker that is used to measure the growth rate of cancer cells, and a high percentage (over 30%) for Ki-67 means that the cancer is likely to grow and spread more quickly.

Prostate cancer (PCa) is a malignancy that may develop into metastatic disease and is most common in men older than 60 years of age [2,3]. Diagnosing PCa based on microscopic biopsy images is challenging, and its accuracy may vary from pathologist to pathologist depending on their expertise and other factors, including the absence of precise and quantitative classification criteria. Diagnosis of PCa is carried out using physical exams, laboratory tests, imaging tests, and biopsies. Core needle biopsy is a technique performed by inserting a thin, hollow needle into the prostate gland to remove a sample of tissue containing many cells.

The image analysis of histological sections holds promise for cancer diagnosis and monitoring disease progression. Compared with Western PCa patients, Korean patients exhibit high scores for risk factors such as high Gleason scores and increased prostate volume. Gleason grading is an important metric in PCa. This system is used to evaluate the prognosis of men with PCa using samples from a prostate biopsy [4]. Cancers with a Gleason score of 6 are considered well-differentiated or low-grade and are likely to be less aggressive. Cancers with Gleason scores of 8–10 are considered poorly differentiated or high-grade and are likely to be more aggressive. It has been reported that PCa is the fifth-most common cancer in males in South Korea and the second-most frequently diagnosed cancer in the world. The incidence of PCa has increased significantly more rapidly in men under 70 years of age than in men over 70 years old [5].

A statistical approach, especially the GLCM texture analysis method, is very common in a medical image analysis and processing system. Textures are generally random and possess consistent properties. Various features based on gray-level intensity computed from a digital image can be used to describe statistical properties such as entropy, contrast, correlation, homogeneity, energy, dissimilarity. Another way to analyse image texture is the use of the frequency domain because it contains information from all parts of the image and is useful for global texture analysis. In order to extract meaningful information from an image, wavelet transformation was performed in this paper for texture analysis.

**Figure 1:**
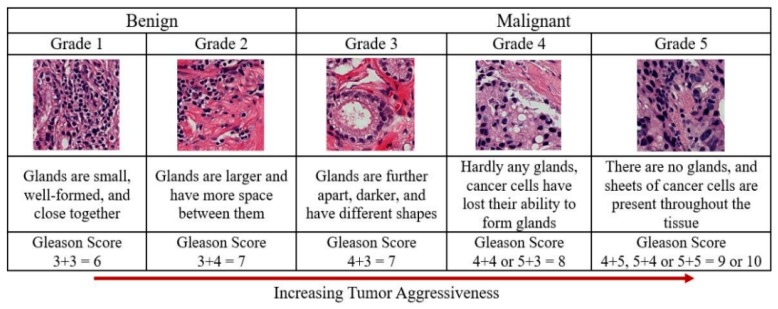
Gleason scores and prostate cancer grading system. The Gleason score for cancer grading is computed by adding the primary and secondary scores from a whole slide H&E-stained microscopic biopsy image. The cancer detection process in histopathology consists of categorising stained microscopic biopsy images into benign and malignant. The Gleason score predicts the aggressiveness of prostate cancer. The images used for Grade 1–5 were scanned at 40× optical magnification, respectively.

Feature extraction is very important when performing cancer grading using biopsy images. Generally, the classification of PCa grading is carried out based on morphological, texture, nuclei cluster, architectural, and colour moment features. However, the current study focuses on wavelet transformation and colour histogram analysis for stained biopsy tissue image processing. At present, automated computerised techniques are in high demand for medical image analysis and processing, and the multilayer perceptron (MLP) is a commonly used technique for feature classification. Texture and colour features are highly significant in tissue image analysis and provide information about the intensity and distribution of colour in an image. The arrangement of the glands and cell nuclei in tissue images and their shape and size differ among Gleason grade groups, shown in Figure 1. The research is presented in five sections. First, a discrete wavelet transform (DWT) was performed on each pathology image using the Haar wavelet function. Second, the grey level co-occurrence matrix (GLCM) was calculated to extract texture features from the wavelet-transformed images. Third, RGB (red/green/blue) colour images were converted and separated into three channels of 8 bits/pixel each. Fourth, the colour distribution of each channel was analysed, and colour moment features were extracted. Fifth, the significant features were selected and classified for predicting cancer grading in histological sections of prostate carcinomas.

## 2. Materials and Methods

Ethical Approval: All subjects’ written informed consent waived for their participation in the study, which was approved by the Institutional Ethics Committee at College of Medicine, Yonsei University, Korea (IRB no. 1-2018-0044). 

### 2.1. Dataset Preparation

Prostate tissue images were obtained from the Severance Hospital of Yonsei University in Seoul, Korea. The size of the whole slide image was 33,584 × 70,352 pixels. These slides were scanned into a computer workstation at 40× optical magnification with a 0.3 NA objective using a digital camera (Olympus C-3000) attached to a microscope (Olympus BX-51), and images were cropped to a size of 512 × 512 pixels and 256 × 256 pixels. We used a total of 400 samples with an image size of 256 × 256 pixels, and each image slice had a resolution of 24 bits/pixel. The collected samples were divided into four groups: benign, grade 3, grade 4, and grade 5. The biopsy tissue was sectioned in 4 μm and the sections were deparaffinized, rehydrated, and stained with hematoxylin and eosin (H&E) using an automated stainer (Leica Autostainer XL). The colour dyes are typically used by pathologists to visualise and analyse tissue components, such as stroma, cytoplasm, nucleus, and lumen.

**Figure A1:**
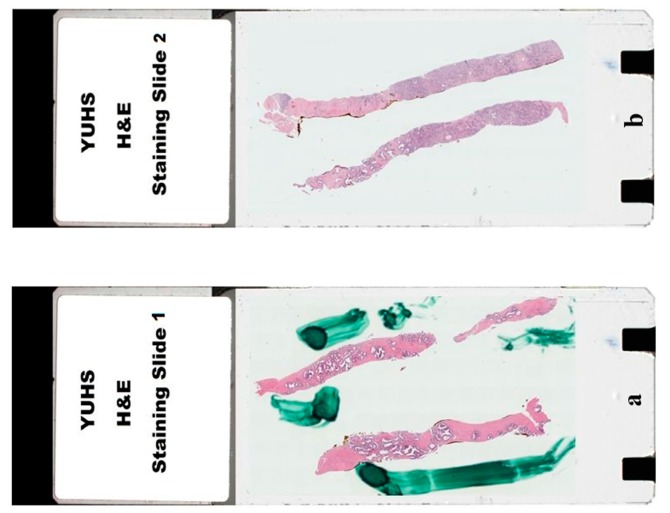
Hematoxilin and Eosin staining slides. (**a**) Staining slide 1 represents grade 3 and grade 4 tissue sections. (**b**) Staining slide 2 represents grade 5 tissue section.

**Figure A2:**
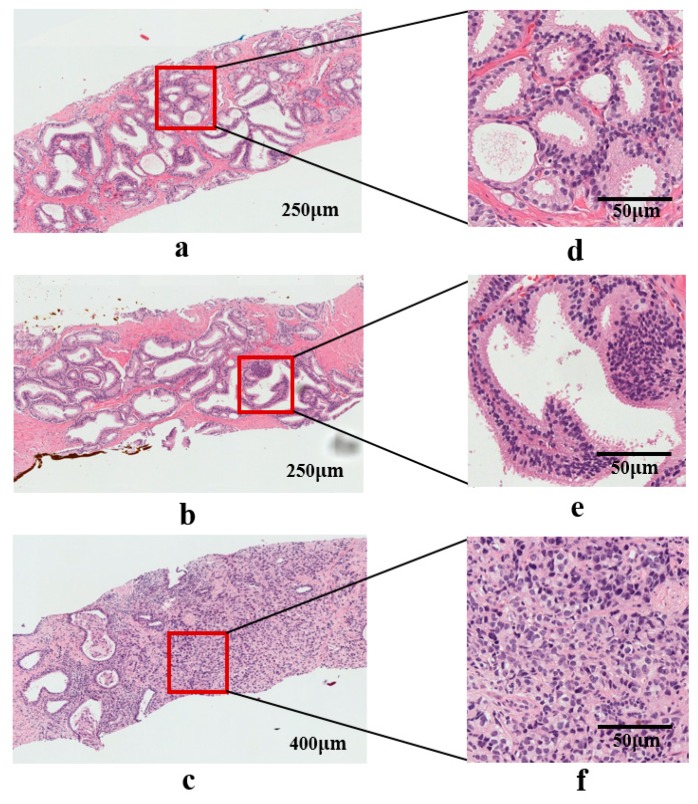
Biopsy tissue images after scanning the slides using a digital camera (Olympus C-3000) attached to a microscope (Olympus BX-51). (**a**–**c**) Whole slide tissue images stained with H&E compounds. (**d**–**f**) Region of interest (ROIs) of grade 3, grade 4, and grade 5, taken from whole slides images (**a**–**c**) respectively.

The H&E stain has been used for over a century and remains essential for identifying different tissue types. This stain reveals a broad range of nuclear, cytoplasmic, and extracellular-matrix features. Briefly, haematoxylin is used in combination with a “mordant”, which is a compound that helps it associate with the tissue. This compound is also called “hematin”, which is positively charged and can react with negatively charged, basophilic cell components, such as nucleic acids in the nucleus. The stain colour for this compound is blue, and the chemical formula is C_16_H_24_O_6_. Eosin is negatively charged and can react with positively charged, acidophilic components, such as amino groups in proteins in the cytoplasm. The stain colour for this compound is pink, and the chemical formula is C_20_H_6_Br_4_Na_2_O_5_. The detail explanation of cell block preparation, tissue processing, and immunocytochemical analysis is in Appendix A. Figure A1 and Figure A2 shows H&E straining slide images and the cropped images extracted from whole slides tissue images, recpectively.

### 2.2. Proposed Pipeline for Analysis and Classification

**Figure 2:**
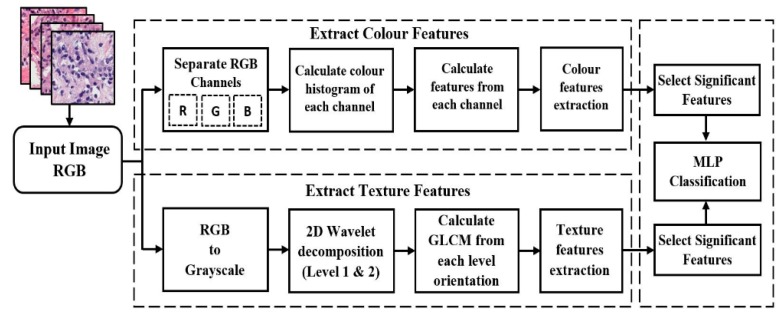
Proposed method for PCa grading in histological sections based on wavelet texture and colour features. Each section is analysed individually and separately for better understanding. Both colour and texture features extraction methods are analysed and performed using grayscale images, which are converted from RGB color images. In the final step, the significant features are selected and classified using an MLP neural network classification algorithm.

MLP classification was performed using a combination of features extracted from microscopic biopsy images of tissue samples. We used a DWT algorithm for wavelet decomposition and computed the GLCM to extract texture features. Colour-feature extraction was also carried out for PCa grading. These features are suitable for the different pathology categories for image analysis chosen for the study. After Haar wavelet transformation, we generated the first and second levels of images and extracted features from each level depth. Artificial neural network (ANN) classification methods were used to classify prostate carcinomas. The proposed methodology in Figure 2 shows the process for extracting colour and texture features from an input image and performing MLP classification using the significant features. MANOVA statistical test was carried out in RStudio development environment, to select the most significant features for classification. This proposed method is expected to improve the classification accuracy rate compared with the method described in our previously published paper. 

### 2.3. Discrete Wavelet Transform

**Figure 3:**
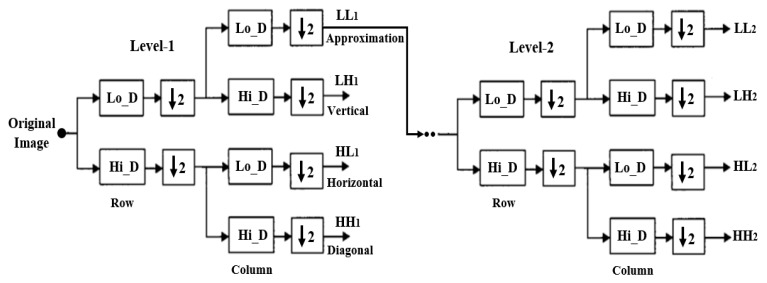
The process of constructing from level-1 to level-2 wavelet transformation. A sequence of two low-pass and high-pass filters, 2Lo_D (row, column) and 2Hi_D (row, column) followed by downsampling provides the approximation and diagonal sub-images, respectively. A combination of low- and high-pass filters and downsamplings provides the vertical and horizontal sub-images.

**Figure 4:**
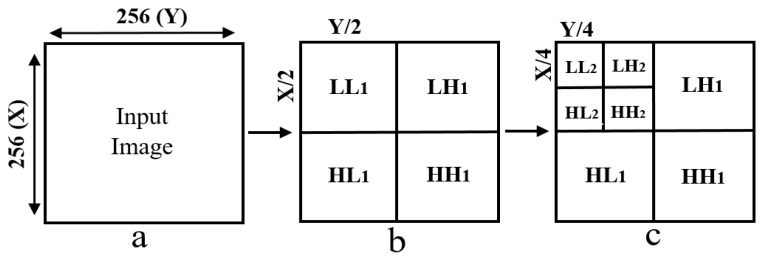
Two-dimensional first- and second-level wavelet decomposition of an input image. (**a**) Input image of size 256 × 256 pixels. (**b**) Structure of level-1 wavelet transform, each sub-image (LL1, LH1, HL1, HH1) has 128 × 128 pixels in size. (**c**) Structure of level-2 wavelet transform, each sub-image (LL2, LH2, HL2, HH2) has 64 × 64 pixels in size.

Wavelet functions are concentrated in time as well as in frequency around a certain point. The wavelet transform is most appropriate for non-stationary signals and basic functions, which vary both in frequency and spatial range. The wavelet transform is designed to provide good frequency resolution for low-frequency components, which are essentially the average intensity values of the image, and high temporal resolution for high-frequency components, which are basically the edges of the image. In the present study, a DWT was performed to divide the information of an image into approximation and detailed sub-signals and extract the most discriminative multi-scale features [6]. In Figure 3, the approximation sub-signal shows the general trend of pixel values, and the detailed sub-signal shows the horizontal, vertical, and diagonal details. Figure 3 shows the two-dimensional first- and second-level wavelet decomposition, in which the original image is the input image for first-level transformation (LL1, LH1, HL1, HH1), and the approximation image (LL1) was used as the input image for second-level transformation (LL2, LH2, HL2, HH2). Figure 4 shows the structure of the first- and second-level wavelet transformation presented in Figure 3, respectively. The input image for the first- and second-level transformations was downsampled by 2, which changed the resolution of the sub-images. The sub-signals of the two-dimensional wavelet decomposition in Figure 4 were computed using the following Equations (1)–(4), respectively [7].

(1) $$W_{\psi }^{A}\left(j,m,n\right)=\frac{1}{\sqrt{MN}}\overset{M-1}{\underset{x=0}{\sum }}\overset{N-1}{\underset{y=0}{\sum }}f\left(x,y\right)\psi_{j_{1},m,n}^{A}$$

(2) $$W_{\phi }^{V}\left(j,m,n\right)=\frac{1}{\sqrt{MN}}\overset{M-1}{\underset{x=0}{\sum }}\overset{N-1}{\underset{y=0}{\sum }}f\left(x,y\right)\phi_{j,m,n}^{V}$$

(3) $$W_{\phi }^{H}\left(j,m,n\right)=\frac{1}{\sqrt{MN}}\overset{M-1}{\underset{x=0}{\sum }}\overset{N-1}{\underset{y=0}{\sum }}f\left(x,y\right)\phi_{j,m,n}^{H}$$

(4) $$W_{\phi }^{D}\left(j,m,n\right)=\frac{1}{\sqrt{MN}}\overset{M-1}{\underset{x=0}{\sum }}\overset{N-1}{\underset{y=0}{\sum }}f\left(x,y\right)\phi_{j,m,n}^{D}$$

where $\psi_{j_{1},m,n}^{A}\left(x,y\right)$ and $\phi_{j,m,n}^{i}\left(x,y\right)$ describe the two-dimensional wavelet functions of level j at pixel in row m and column n of an input image with rows M and columns N number of pixels, A represent an approximation orientation and i represents the other orientation of wavelet details coefficients, namely vertical, horizontal, and diagonal.

#### Haar Wavelet Transform

**Figure 5:**
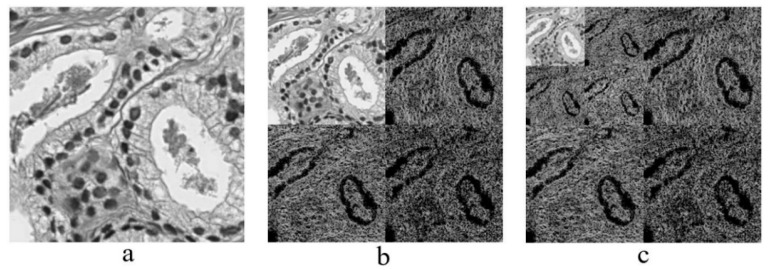
Haar wavelet transformation. (**a**) Original image converted from RGB to grayscale, which was cropped from a whole slide tissue image and scanned at 40× optical magnification. (**b**) Level-1 wavelet decomposition. (**c**) Level-2 wavelet decomposition.

The simplest type of wavelet transform is called a Haar wavelet. It is related to a mathematical operation called the Haar transform. It is performed in several stages, or levels. Haar transform decomposes a discrete signal into two sub-signals of half its length [8,9]. One sub-signal is a running average, and the other sub-signal is a running difference, storing details coefficients, eliminating data, and reconstructing the matrix such that the resulting matrix is similar to the initial matrix. The low-pass (Lo_D) and high-pass (Hi_D) filters are given by $\mathrm{Lo}\text{\_}D=\left[\frac{1}{\sqrt{2}},\;\frac{1}{\sqrt{2}}\right]$ and $\mathrm{Hi}\text{\_}D=\left[\frac{1}{\sqrt{2}},\;-\frac{1}{\sqrt{2}}\right]$ which are basically used for Haar wavelet transformation [10,11]. Figure 5 shows the multilevel wavelet transformed images of prostate carcinomas, and this implementation was carried out in MATLAB R2018a, where a 24-bit RGB color image was converted into 8-bit for processing the image.

**Figure 6:**
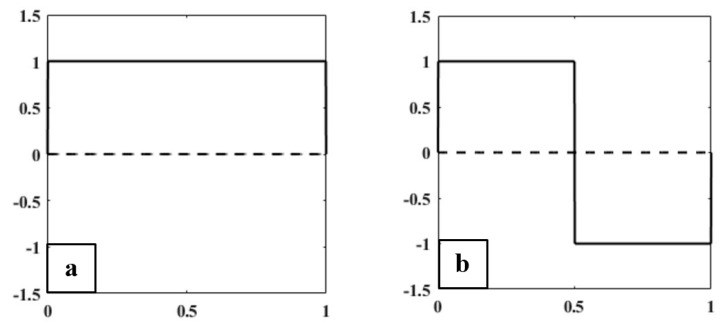
(**a**) Haar scaling function (Father Wavelet). (**b**) Haar wavelet function (Mother Wavelet).

There are two functions that play a primary role in wavelet analysis, the scaling function ϕ (father wavelet) and the wavelet function ψ (mother wavelet), shown in Figure 6. In the Haar wavelet transform, the scaling and wavelet functions can be treated as extractors of particular image features. The scaling and wavelet functions in Figure 6 were constructed based on the unit interval (0 to 1) and (−1 to 1), respectively. Haar scaling and wavelet function can be described as

(5) $$\mathrm{Father}\;\mathrm{Wavelet}\;\phi \left(x\right)=\{\begin{array}{c} \;\;\;1\;\;\;\;\;\;\;for\;0\leq x<1\\ 0\;\;\;\;\;\;\;\;\;\;\;otherwise \end{array}$$

(6) $$\mathrm{Mother}\;\mathrm{Wavelet}\;\psi \left(x\right)=\{\begin{array}{c} \;\;\;\;\;\;1\;\;\;\;\;\;\;for\;0\leq x<1/2\;\\ \;\;-1\;\;\;\;\;\;for\;1/2\leq x<1\;\\ \;\;0\;\;\;\;\;\;\;\;\;\;\;\;\;\;otherwise \end{array}$$

### 2.4. Color Moment Analysis

**Figure 7:**
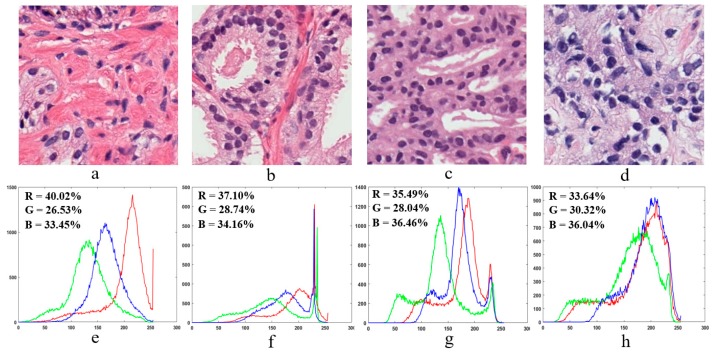
Sample microscopic biopsy images of prostate tissue. (**a**–**d**) Prostate tissue images of benign, grade 3, grade 4, and grade 5 based on the Gleason grading system, respectively. These images were scanned at 40× optical magnification. (**e**–**h**) Colour histograms in RGB colour space are generated from the tissue images in (**a**–**d**), respectively.

In colour moment analysis, a colour histogram is used to represent the colour distribution in microscopic biopsy tissue images. Colour information is a very important factor for tissue image analysis, and each peak of the histogram represents a different colour, as shown in Figure 7. In Figure 7e–h, the x-axis and y-axis of the colour histogram show the number of bins and colour pixels present in an image, respectively. Colour moment analysis was carried out to extract colour-based features from prostate tissue images [12].

Colour moment analysis is essential in digital medical image processing to carry out colour image enhancement or information retrieval. Figure 7e–h shows the colour information of each individual channel present in the prostate tissue images of Figure 7a–d, respectively. We computed the percentage of each individual colour in the RGB channel using the following formula:

(7) $$\mathrm{Colour}\;\mathrm{Percentage}\;\left[\%\right]=\frac{Total\;number\;colour\;channnel\;pixels}{Total\;number\;of\;image\;pixels}\times 100$$

### 2.5. Feature Extraction

Two-dimensional (2D) grey-level co-occurrence matrix (GLCM) and colour moment techniques were used to extract texture and pixel-based colour moment features, respectively. Texture features were extracted from Haar wavelet-transformed images, and colour features were extracted from 8-bit grayscale images that were separated into three channels from an original colour image. We extracted a total of 11 features and carried out a multivariate analysis of variance (MANOVA) statistical test to identify the significant features [13,14,15,16].

#### 2.5.1. Texture Features

**Figure 8:**
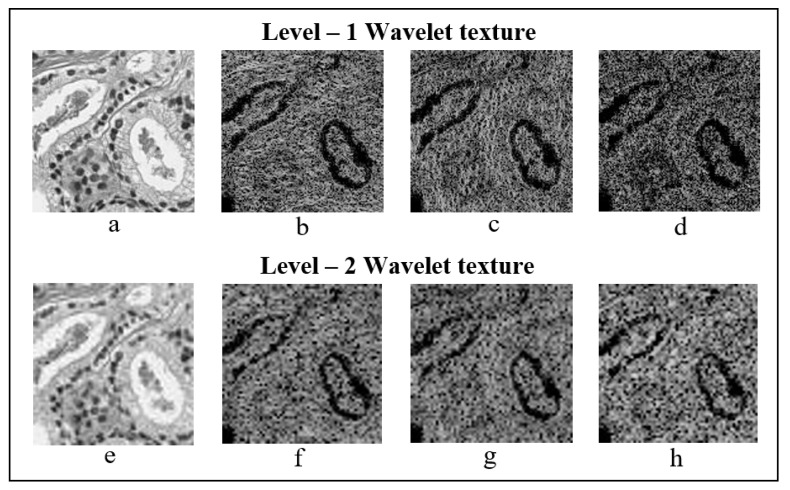
Level-1 and level-2 Haar wavelet texture images. (**a**–**d**) First-level orientation sub-images, each 128 × 128 pixels in size. (**e**–**h**) Second-level orientation sub-images, each 64 × 64 pixels in size. From level-1 and level-2, (**a**,**e**), (**b**,**f**), (**c**,**g**) and (**d**,**h**) represents the wavelet coefficients, which includes approximation, vertical, horizontal, and diagonal, respectively.

GLCM is a statistical method that examines texture based on the spatial relationship of pixels. To analyse image texture, we first created a GLCM to calculate how often pairs of pixels with specific values and in a specified spatial relationship occurred in an image, and then extracted statistical measures from the matrix. The directions of the co-occurrence matrix for feature extraction were as follows: 0° [0, 1], 45° [−1, 1], 90° [−1, 0], and 135° [−1, −1]. So, the values of GLCM matrix are always within the range [0, 1]. The size of the GLCM matrix used for texture analysis was 128 × 128 and 64 × 64, for first- and second-level sub-images, respectively. GLCM texture features were separately extracted after wavelet transformation at level-1 and level-2 from each orientation, shown in Figure 8. Based on the GLCM, we computed six different types of wavelet texture features: contrast, homogeneity, correlation, energy, entropy, and dissimilarity [17,18,19,20]. These features were calculated using the following equations:

(8) $$Contrast=\overset{N-1}{\underset{i=0}{\sum }}\overset{N-1}{\underset{j=0}{\sum }}{\left|i-j\right|}^{2}p\left(i-j\right)$$

(9) $$Homogeneity=\overset{N-1}{\underset{i=0}{\sum }}\overset{N-1}{\underset{j=0}{\sum }}\frac{p\left(i,j\right)}{1+{\left(i+j\right)}^{2}}$$

(10) $$Correlation=\overset{N-1}{\underset{i=0}{\sum }}\overset{N-1}{\underset{j=0}{\sum }}p\left(i,j\right)\frac{\left(i-\mu_{x}\right)\left(i-\mu_{y}\right)}{\sigma_{x}\sigma_{y}}$$

(11) $$Energy=\overset{N-1}{\underset{i=0}{\sum }}\overset{N-1}{\underset{j=0}{\sum }}p{\left(i,j\right)}^{2}$$

(12) $$Entropy=-\overset{N-1}{\underset{i=0}{\sum }}\overset{N-1}{\underset{j=0}{\sum }}p\left(i,j\right)\;\log\left(p\left(i,j\right)\right)$$

(13) $$Dissimilarity=\overset{N-1}{\underset{i=0}{\sum }}\overset{N-1}{\underset{j=0}{\sum }}\left|i-j\right|p\left(i-j\right)$$

where *p*(*i* – *j*) signifies the co-occurrence probability matrix for a combination of two pixels with intensity (*i*, *j*) that occur in an image, separated by a given distance. *N* signifies the quantized grey level, and *µ_x_* and *µ_y_* are the means, and $\sigma_{x}$ and $\sigma_{y}$ the standard deviations, for row *i* and column *j*, respectively, within the GLCM.

#### 2.5.2. Colour Moment Features

**Figure 9:**
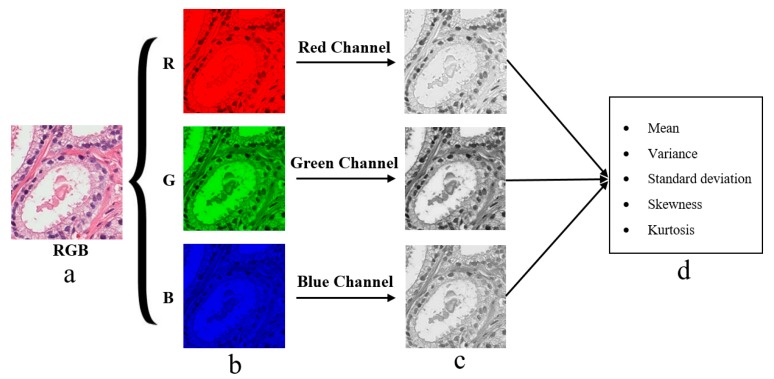
Tissue image processing and colour moment analysis by splitting RGB colour channels. (**a**) Original RGB tissue image stained with H&E compunds. (**b**) The red, green and blue component converted from an original image (**a**) with the resolution of 24-bits/pixel. (**c**) The respective split channels of R, G and B images present in (**b**). Specifically, these images are formed by converting each 24-bit R, G and B images into 8-bit grayscale images, respectively. (**d**) The extracted features from each color channel present in (**c**), respectively.

The pixel-based colour moment descriptor (PCMD) technique was used to extract colour-based features from prostate tissue images. This technique is useful for analysing the colour distribution among the three different channels of RGB colour images. To extract useful colour moment features from tissue images, the three colour channels were separated from RGB colour images, as shown in Figure 9. We then computed the mean, standard deviation, skewness, variance, and kurtosis from each individual channel separately [21,22]. These features were calculated using the following equations:

(14) $$Mean\;\left(\mu_{i}\right)=\overset{N}{\underset{j=1}{\sum }}\frac{1}{N}p_{ij}$$

(15) $$Standard\;deviation\;\left(\sigma_{i}\right)=\sqrt{\left(\frac{1}{N}\overset{1}{\underset{N}{\sum }}{\left(p_{ij}-\mu_{i}\right)}^{2}\right)}$$

(16) $$Skewness\;\left(s_{i}\right)=\sqrt[{3}]{(\frac{1}{N}\overset{1}{\underset{N}{\sum }}{\left(p_{ij}-\mu_{i}\right)}^{3}})$$

(17) $$Kurtosis\;\left(k_{i}\right)=\sqrt[{4}]{(\frac{1}{N}\overset{1}{\underset{N}{\sum }}{\left(p_{ij}-\mu_{i}\right)}^{4}})$$

where, $p_{ij}$ is the value of $j^{th}$ pixel of the image at the $i^{th}$ color channel. N is the number of pixels in the image. $\mu_{i}$ is the mean value, $\sigma_{i}$. is the standard deviation and it is obtained by taking the square root of the variance of the color distribution, $s_{i}$ is the skewness, and $k_{i}$ is the kurtosis.

### 2.6. MLP Classification

MLP is a supervised classification system that consists of at least three layers of nodes: the first layer is the input layer, the middle layer is the hidden layer, and the last layer is the output layer. Input and output layers are used to feed in data and obtain the output results, respectively [23,24,25,26]. However, the hidden layer can be modified to increase the complexity of the model. It is a feed-forward ANN, in which each node is a neuron of hidden and output layers and uses a nonlinear activation function. To carry out PCa grading, binary classification was performed using the MLP neural network classifier in Waikato Environment (WEKA), which classified the samples as benign vs. malignant, benign vs. grade 3, benign vs. grade 4, benign vs. grade 5, and grade 3 vs. grade 4,5. WEKA was developed at the University of Waikato, New Zealand. It supports many data mining tasks, such as classification, clustering, regression, and feature selection [27].

**Figure 10:**
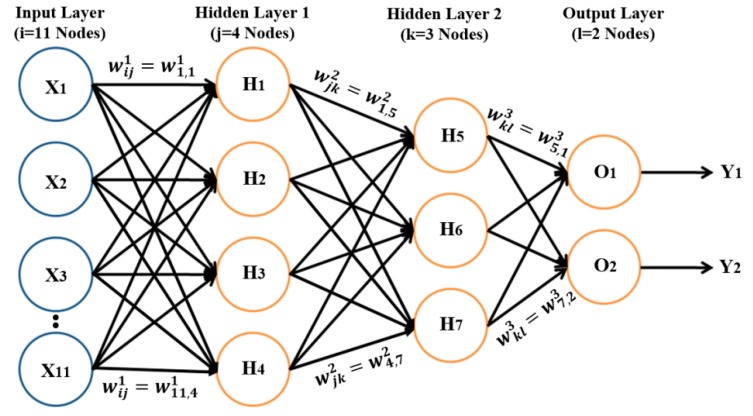
MLP classifier model with input, hidden, and output layers. The input layer contains 11 nodes, the output layer contains 2 nodes, and hidden layers 1 and 2 contain 4 and 3 nodes, respectively. The perceptron takes 11 input features (X_1_–X_11_) extracted from prostate tissue images, and weights (W_1_–W_3_) are associated with those inputs. The network performs a weighted summation to produce an output Y, and perceptron weight is determined during the training process based on training data.

To train the model, we input data into the model, multiplied the data with the weights, and obtained the computed output of the model, which is called a forward pass. We also carried out backward passes, in which model weight was updated based on the calculated loss from the expected and predicted outputs. The learning rate is a hyperparameter in the neural network that controls how the model is changed in response to the estimated error each time the model weight is updated based on the learning rate and momentum. Momentum is a technique that frequently improves both training speed and accuracy [28,29]. The activation function used for the training process was the sigmoid function, which takes an input value and squashes it to within the range of 0 and 1, to perform binary classification. We designed the network with one input layer, two hidden layers, and one output layer, shown in Figure 10. The weight was updated automatically while training the model based on the following equations:

(18) w=w+learning rate∗(expected−predicted)∗n

(19) $$z=\overset{n}{\underset{i=1}{\sum }}wixi+bias\;\mathrm{Sigmoid}\;\mathrm{Function}:\;f\left(z\right)=\frac{1}{1+exp^{-z}}$$

where w is the weight, n is the number of features in x, and z is the linear form of equation used in the sigmoid function, to generate non-linear activation function.

## 3. Results and Discussion

In the present study, we extracted wavelet-based texture and colour moment features for MLP neural network classification. Images were 256 × 256 pixels (24 bits/pixel) in size and were converted to 8 bits/pixel for wavelet transformation (first- and second-level), colour moment analysis, and feature extraction. A total of 400 images were used for PCa grading classification and were divided equally among four classes: benign, grade 3, grade 4, and grade 5. A ratio of 7:3 was fixed for training and test data sets, respectively. The algorithms used for image transformation, feature extraction, and classification were implemented in MATLAB R2018a and the WAIKATO environment [30,31]. We selected the most significant features based on *F*-values and *P*-values obtained from MANOVA statistical tests performed using the R programming language in RStudio. Table 1 and Table 2 show the results of binary MLP classification for the five different groups of prostate carcinomas.

### Performance Metrics

We used a number of metrics to evaluate the performance of the classification model and deep learning algorithm, including accuracy, sensitivity, specificity, F1-score, and Matthew’s correlation coefficient (MCC). The four types of confusion matrices used for computing performance metrics were true positive (TP), true negative (TN), false positive (FP), and false negative (FN). The following metrics were used:

1. **Accuracy:** How many TP and TN were obtained out of all outcomes among all samples.
  (20) $$Accuracy=\frac{TP+TN}{TP+TN+FP+FN}\times 100$$
2. **Sensitivity:** The rate of correctly classifying samples positively.
  (21) $$Sensitivity=\frac{TP}{TP+FN}\times 100$$
3. **Specificity:** The rate of correctly classifying samples negatively.
  (22) $$Specificity=\frac{TN}{TN+FP}\times 100$$
4. **F1-score:** It is calculated using a combination of the “Precision” and “Recall” metrics. The precision metric indicates how many classified samples are relevant, and the recall metric represents how many relevant samples are classified.
  (23) $$F1-score=2\times \frac{Precision\times Recall}{Precision+Recall}\times 100$$
5. **MCC:** An index of the performance binary of classification. Indicates the correlation between the observed and predicted binary classification.
  (24) $$MCC=\frac{TP\;\times \;TN\;-\;FP\;\times \;FN}{\sqrt{\left(\left(TP\;+\;FN\right)\left(TP\;+\;FP\right)\left(TN\;+\;FN\right)\left(TN\;+\;FP\right)\right)}}\times 100$$

**Figure 11:**
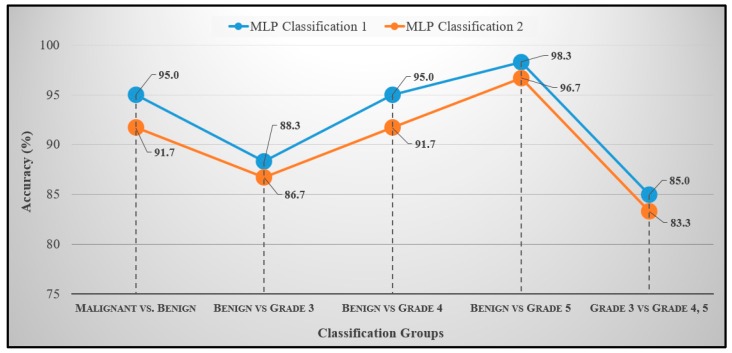
Classification accuracy for the five binary divisions. Each group was classified separately and independently to minimise the error rate and increase the performance of the classification. MLP Classification 1 represents accuracy rates based on level-1 wavelet-based texture and colour features, and MLP Classification 2 represents accuracy rates based on level-2 wavelet-based texture and colour features.

The accuracy rates listed in Table 1 and Table 2 were used to construct line graphs, and the results were compared between the two classification modes, shown in Figure 11. The data that can viewed in the line graph are the test accuracies of MLP neural network classification. The blue and orange graph in Figure 11 shows the classification performance between Table 1 and Table 2, respectively. It is clear from these results that the wavelet image features of level-1 were more reliable than those of level-2. MLP binary classification was carried out among five groups, and each group was classified independently and separately.

**Figure 12:**
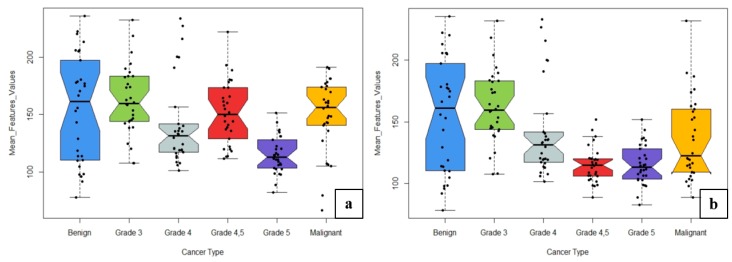
Comparison between the two levels of classification using wavelet texture and colour moment features. (**a**) Features used for classification 1. (**b**) Features used for Classification 2. Upper and lower whiskers are the maximum and minimum mean feature values, respectively, and the central line is the median value (50th percentile). Dots represent the mean values for each cancer type.

The learning rate and momentum for each group classification changed based on the accuracy of the training and test dataset. The weighted average accuracy rates obtained from MLP Classifications 1 and 2 were 92.7% and 90.0%, respectively. The combination of texture and colour moment features in MLP Classification 1 produced the most accurate results using the neural network MLP technique. In neural network classification, the network parameters “Learning Rate” and “Momentum” were adjusted several times to accurately construct the network architecture and increase the accuracy for the test dataset. Figure 12 shows the box plots for six different classes used for MLP Classifications 1 and 2. A total of 11 features were used for histological grade classification. To construct the box plots, we used the weighted mean values for all features based on six classes: benign, grade 3, grade 4, grade 5, grades 4,5 and malignant.

As shown in Figure 12a,b, the box plots of different grades varied in size based on the mean feature values. We used the test dataset to construct the box plots and compare the results between grades. The benign grade was more likely to have higher mean values than other grades. Thus, grade 3, grade 4, grade 5, and malignant classifications could be easily distinguished from benign tumours. Similarly, classification was performed for grade 3 vs. grades 4,5, but it was difficult to distinguish between them. It can be concluded that the wavelet texture features based on GLCM and colour moment features can accurately differentiate among six different classes in images of histological sections. The methodology proposed in this paper combines wavelet texture, colour feature extraction, and MLP classification, which produced the most accurate classification of prostate carcinomas. Classification using MLP techniques requires more time for data processing and optimization because the learning rate, momentum and number of hidden layers must be adjusted based on training and test accuracy. The use of the wavelet transform technique for analysis of microscopic biopsy images includes the following advantages:

(a) It provides concurrent localisation in time and frequency domains.
(b) Small wavelets can be used to separate fine details in an image, and large wavelets can identify coarse details.
(c) An image can be de-noised without appreciable degradation.

Haar and Daubechies wavelets are usually used to extract texture features, detect multi-resolution characteristics, and for texture discrimination and pattern recognition. However, in the current study, we used Haar wavelet features extracted from the detailed coefficients of the transformed image at levels 1 and 2. These features revealed the characteristics of the image and were used for classifying grades of cancer observed in histological sections. Texture analysis has been an important topic in image processing and computer vision. This method is an important issue in many areas like object recognition, image retrieval study, medical imaging, robotics and remote sensing. RGB colour images were used to extract colour moment features from each channel depth. Colour texture extraction is critically important for microscopic biopsy image analysis of histological tissue sections. Texture is a connected set of pixels that occurs repeatedly in an image, and it provides information about the variation in the intensity of a surface by quantifying properties such as smoothness, coarseness, and regularity. Therefore, we performed an MLP classification using a combination of colour and wavelet texture features. The performance measures we used to evaluate the classification results included accuracy, sensitivity, specificity, F1-score, and MCC.

Our approach has been distinguishing different grade groups of prostate carcinoma: benign, grade 3, grade 4, and grade 5. In PCa grading system, grade 1 and grade 2 are classified as a benign/normal tumor, whereas grades 3, 4, and 5 are classified as a malignant/abnormal tumor. The Gleason grade groups, we used for MLP neural network classification are: “Benign vs. Malignant”, “Benign vs. Grade 3”, “Benign vs. Grade 4”, “Benign vs. Grade 5”, and “Grade 3 vs. Grades 4,5”. We have extracted six texture features (contrast, homogeneity, correlation, energy, entropy, and dissimilarity) and five colour moment features (mean, standard deviation, variance, skewness, and kurtosis), from 40× optical magnification biopsy images. After going through this research, it is clear that an image texture carries useful diagnostic information, which is very essential for medical image processing and discrimination with benign and malignant tumors. However, the proposed features are not sufficient to identify and discriminate between benign and malignant; it is necessary to identify and discriminate them with more reliable features. We have compared our proposed approach with the other methods to show the performance accuracy of each method achieved by using different types of features and classification groups, shown in Table 3.

Hae-Gil et al. [8] analysed the texture features of Haar- and Daubechies-transformed wavelets in breast cancer images. Tissue samples were analysed from ductal regions, and included benign ductal hyperplasia, ductal carcinoma in situ (DCIS), and invasive ductal carcinoma (CA). The classification was carried out on up to six levels of wavelet images using discriminant analysis and neural network methods. The highest classification accuracy (87.78%) was obtained using second-level Haar wavelet images. Kourosh et al. [10] used the co-occurrence matrix and wavelet packet techniques for computing textural features. They used 100 colour images of tissue samples of PCa graded 2–5. The images were of different sizes and magnification 100×. Wavelet transformation was carried out for the first and second levels of decomposition. The classification was performed using the k-nearest neighbour (k-NN) algorithm and achieved a maximum accuracy of 97%. Mahmut et al. [11] proposed ANN methods for the classification of cancer and non-cancer prostate cells. Gauss Markov random fields (GMRF), Fourier entropy, and wavelet average deviation features were calculated from images of 80 non-cancerous and cancerous prostate cell nuclei. For classification, ANN techniques including MLP, radial basis function (RBF), and learning vector quantization (LVQ), were used. The MLP technique included two models (MLP1 and MLP2). MLP2 achieved the highest classification rate among all classifiers (86.88%). Issac et al. [12] employed complex wavelets for multiscale image analysis to extract a feature set for the description of chromatin texture in the cytological diagnosis of invasive breast cancer. They used a dataset consisting of a total of 45 images, 25 of which were benign and 20 malignant. The obtained feature sets were used for classification using the k-NN algorithm, and an average accuracy of 93.33% was achieved. Tai et al. [15] proposed a nobel method to classify prostatic biopsy according to the Gleason grading system. They extracted wavelet-based fractal dimension features from each sub-band, and carried out SVM classification. The experimental result they obtained was 86.3% accuracy for 1000 pathological images. Naik et al. [16] presented a method for automated histopathology images. They have demonstrated the utility of glandular and nuclear segmentation algorithm in accurate extraction of various morphological and nuclear features for automated grading of prostate cancer, breast cancer, and distinguishing between cancerous and benign breast histology specimens. The authors used an SVM classifier for classification of prostate images containing 16 Gleason grade 3 images, 11 grade 4 images, and 17 benign epithelial images of biopsy tissue. They achieved an accuracy of 95.19% for grade 3 vs. grade 4, 86.35% for grade 3 vs. benign, and 92.90% for grade 4 vs. benign. Nguyen et al. [19] introduced a novel approach to grade prostate malignancy using digitised histopathological specimens of the prostate tissue. They have extracted tissue structural features from the gland morphology and co-occurrence texture features from 82 regions of interest (ROI) with 620 × 550 pixels to classify a tissue pattern into three major categories: benign, grade 3 carcinoma, and grade 4 carcinoma. The authors proposed a hierarchical (binary) classification scheme and obtained 85.6% accuracy in classifying an input tissue pattern into one of the three classes. Diamond et al. [20] extracted Haralick texture and morphological features, to classify sub-regions in a prostate tissue image. They achieved an accuracy of 79.3% when evaluating the algorithm on sub-regions of 8 tissue images. Albashish et al. [32] proposed a number of texture features, including Haralick, histogram of oriented gradient (HOG), and run-length matrix, which were individually extracted from images of nuclei and lumens. An ensemble machine-learning classification system achieved an accuracy of 92.4% for benign vs. grade 4 and 97.85% for benign vs. grade 3 classifications. Doyle et al. [33] also used image texture features to perform pixel-wise Bayesian classification at each image scale to obtain the corresponding likelihood scenario. The authors achieved an accuracy of 88% for distinguishing between benign and malignant samples. Shaukat et al. [34] developed a computer-aided diagnosis system for the automatic grading of histological images of PCa tissue. Their system is based on 2D discrete wavelet packet decomposition and SVM. They used the Haar wavelet filter for wavelet decomposition up to level 3 for calculating the GLCM texture features of sub-images. A total of 129 images were used for classification, of which 43 were grade 3, 44 grade 4, and 42 grade 5, and they achieved an average accuracy of 92.24% across all three classes. Kim et al. [35] performed texture analysis using GLCM method and carried out classification through machine learning techniques, namely SVM and k-NN. The classification was performed based on 10 features (using ANOVA) and 12 features (without using ANOVA), separately. The highest accuracy of 90% was achieved by SVM for benign vs. grade 4,5. In our previous study [36], we discussed the morphological analysis of the cell nucleus and lumen and performed k-means colour segmentation and watershed segmentation to identify regions of interest in tissue images and isolate the cell nucleus, respectively. We used patch images that were 512 × 512 pixels (24 bits/pixel) in size that were cropped from an original whole slide tissue image that was 33,584 × 70,352 pixels in size. The classification was carried out using SVM and achieved an average accuracy of 90.2%.

From the above related works, it can be analysed that few authors used multi- and binary-classification. In this paper, our proposed approach is binary MLP neural network classification. Among all the authors described in Table 3, Hae-Gil et al. (2005) used breast cancer tissue images for wavelet analysis and performed multiclass classification, whereas we focused on prostate images for colour and wavelet analysis and performed binary classification. We compared this published method with our approach to check the performance between breast and prostate cancer grading classification.

Among all the binary classification approaches mentioned in Table 3, our proposed method has obviously outperformed other published works with the exemption of Albashish et al. (2017) whose benign vs. grade 3 resulted in 97.9% accuracy. Nevertheless, this result is not comparable to our approach as their feature extraction methods were different. Moreover, the change in accuracy depends on the features being extracted as well as the type of classification methods being used for detecting prostate carcinomas in histological sections.

## 4. Conclusions

In conclusion, biopsy tissue images are widely used for PCa diagnosis and to determine the level of malignancy for cancerous tissue. To determine the histological grades of PCa, biopsied tissue is stained with H&E and viewed by pathologists under a microscope. By applying DWT to predict the cancer grades in biopsy images, we were able to capture relevant information from the sub-band transformed images. Similarly, the colour moment technique can be used to extract the different types of colour information present in histological biopsy images. In the current study, a colour histogram was used to visualise the colour variation in tissue images. Our study shows that GLCM and PCMD methods can be used to extract significant features from histological images of prostate carcinomas. Haar wavelet transformation was carried out up to level-2, and texture features were separately extracted from sub-images at each level depth.

Paraffin cell block preparation, tissue processing and paraffin embedding, and immunocytochemical analysis were carried out in the Severance Hospital of the Yonsei University, South Korea. It is very important to carry out de-paraffinization and rehydration process for staining the tissue slides using H&E chemical compounds.

The present study employed an ANN method, and classification was performed using an MLP classifier. Wavelet-based texture and colour features have the potential to be used in the analysis of microscopic biopsy images of histological sections. According to the proposed model in Figure 2, we performed all the necessary steps for biopsy image analysis, feature extraction, and classification, which worked well with prostate tissue images and achieved promising results. Automated computerised systems for cancer classification will greatly help pathologists in cancer diagnosis and grading.

## Figures and Tables

**Table 1:** MLP classification performance based on level 1 wavelet texture and colour moment features.

Groups	Accuracy (%)	Sensitivity (%)	Specificity (%)	F1-Score (%)	MCC (%)
Benign vs. Malignant	95.0	96.5	93.5	94.9	90.0
Benign vs. Grade 3	88.3	82.9	96.0	89.2	77.7
Benign vs. Grade 4	95.0	93.5	96.5	95.1	90.0
Benign vs. Grade 5	98.3	100.0	96.8	98.3	96.7
Grade 3 vs. Grade 4/5	85.0	95.4	76.9	80.8	69.5

**Table 2:** MLP classification performance based on level 2 wavelet texture and colour moment features.

Groups	Accuracy (%)	Sensitivity (%)	Specificity (%)	F1-Score (%)	MCC (%)
Benign vs. Malignant	91.7	93.1	90.3	91.5	83.4
Benign vs. Grade 3	86.7	80.6	95.8	87.9	74.8
Benign vs. Grade 4	91.7	90.3	93.1	91.8	83.4
Benign vs. Grade 5	96.7	96.7	96.7	96.7	93.3
Grade 3 vs. Grade 4/5	83.3	95.4	76.3	80.8	69.2

**Table 3:** Comparison of published methods with the proposed method for tissue characterisation. Multi-class and binary-class classifications were performed by the authors using different types of features, which were classified using machine learning techniques listed in a given table. SVM, support vector machine; k-NN, k-nearest neighbour; MLP, multilayer perceptron; GMRF, Gauss Markov random fields; HOG, histogram of oriented gradients; GLCM, gray level co-occurrence matrix; DCIS, ductal carcinoma in situ; CA, invasive ductal carcinoma.

Authors	Method	Features	Classes	Accuracy
Hae-Gil et al., 2005 [8]	Discriminant analysis	Wavelet features (Haar, level-2)	Benign, DCIS, CA	87.8%
Kourosh et al., 2003 [10]	k-NN	Multiwavelet texture features	Grades 2, 3, 4, 5	97.0%
Mahmut et al., 2007 [11]	MLP	Texture (GMRF, Fourier entropy, wavelet)	Benign vs. Malignant	86.9%
Issac et al., 2010 [12]	k-NN	Wavelet texture features	Benign vs. Malignant	93.3%
Tai et al., 2010 [15]	SVM	Wavelet-based fractal dimension	Normal, Grade 3, 4, 5	86.3%
Naik et al., 2008 [16]	SVM	Shape features of the lumen and the gland inner boundary	Grade 3 vs. Grade 4	95.2%
			Benign vs. Grade 3	86.3%
			Benign vs. Grade 4	92.9%
Nguyen et al., 2012 [19]	SVM	Gland morphology and co-occurrence	Benign, Grade 3 and 4 carcinoma	85.6%
Diamond et al., 2004 [20]	Machine vision assessment	Colour, texture, and morphometric	Stroma, benign tissue and prostatic carcinoma	79.3%
Albashish et al., 2017 [32]	SVM	Texture features, (Haralick, HOG, run-length matrix)	Grade 3 vs. Grade 4	88.9%
			Benign vs. Grade 3	97.9%
			Benign vs. Grade 4	92.4%
Doyle et al., 2006 [33]	Bayesian	Texture features, (first-order statistics, co-occurrence matrix, wavelet)	Benign vs. Malignant	88.0%
Shaukat et al., 2016 [34]	SVM	Wavelet texture features	Grades 3, 4, 5	92.2%
Kim et al., 2019 [35]	SVM	GLCM co-occurrence matrix	Benign vs. Malignant	84.1%
			Grade 3 vs. Grade 4,5	85.0%
Subrata et al., 2019 [36]	SVM	Morphological features	Benign vs. Malignant	88.7%
			Grade 3 vs. Grades 4,5	85.0%
			Grade 4 vs. Grade 5	92.5%
			Grade 3	90.0%
			Grade 4	90.0%
			Grade 5	95.0%
Proposed	MLP	Wavelet texture (level-1) and colour features	Benign vs. Malignant	95.0%
			Grade 3 vs. Grade 4,5	85.0%
			Benign vs. Grade 3	88.3%
			Benign vs. Grade 4	95.0%
			Benign vs. Grade 5	98.3%

## Appendix A

FFPE (Formalin-Fixed, Paraffin-Embedded) is a commonly used format for storing solid tissue pathology specimens, especially tumor samples. In order to carry out H&E staining procedure, the slides must be de-paraffinized and rehydrated. Deparaffinization procedure is performed to remove the paraffin wax from slides prior to staining, and the incomplete removal of paraffin can lead to poor staining of the tissue section.

**To carry out paraffin cell block preparation:**

(a) Centrifuge the samples and transfer 200 to 400 µL (microliter) supernatant plasma aliquots into an individual microfuge tube.
(b) Add 200 µL of plasma, about 200 µL of thromboplastin, and about 200 µL of 0.025 molar calcium chloride to the fixed cultured cells and allow the mixtures to form cell clots at room temperature for about ten minutes.
(c) Wash the cell clots 2× with 1 mL of PBS (phosphate buffered saline), fully transferring the clots onto individual pieces of formalin moistened filter paper after the second wash.
(d) Wrap the clots in the filter paper and use a pin set to place the clots to an individual tissue holder in the center of four other pieces of formalin moistened paper.
(e) Place the tissue holder into a glass jar containing 50 mL of buffered formalin for overnight formalin fixation at 4 °C.

**To carry out tissue processing and paraffin embedding:**

(a) Load the tissue holder into a tissue processor that was kept in a glass jar for overnight formalin fixation.
(b) At least one hour before the end of the processing procedure, turn on a heated embedding station to melt the paraffin.
(c) When the embedded station and the clot are ready, confirm the presence of molten paraffin in the metal mold and transfer a formed cell clot in the paraffin.
(d) Place a new tissue case without the lid into the metal mold and cover the case with more molten paraffin.
(e) Let the paraffin solidify in a cold plate for 30 to 60 s. Then separate the tissue case from the metal mold.

**To prepare sections for immunocytochemical analysis:**

(a) Locate the cell clot in one paraffin cell block and use a microtome to cut the block into 3–4 micrometer (µm) thick slices.
(b) Place paraffin sections onto saline coated glass slides and place the slides into a 37 °C oven for 30 min.
(c) When the sections have adhered to the slides, de-paraffinize the slides in 15 mL of xylene for 3–4 min followed by dehydration of the sections with sequential 2 min in ethanol incubations.
(d) After the 80% ethanol incubation, wash the sections in running water for 10 min to remove the ethanol and boil the slides in a jar containing 40 mL of Tris-EDTA retrieval buffer for 30 min.
(e) At the end of the incubation, wash the antigen retrieved slides under running water followed by a 10 min incubation in 95% ethanol at 4 °C.
(f) Wash the slides in TBS-T (Tris Buffered Saline) followed by incubation in hydrogen peroxide block for 15 min to remove any remnant peroxidase activity.
(g) Wash the slides three times in TBS-T each for two minutes, then label the sections with 100 mL of primary antibody mixture from the immunocytochemical stain kit of interest for one hour followed by TBC-T washes for five times, each for two minutes.
(h) After the last wash, incubate the slides in primary antibody enhancer from the kit for 15 min at room temperature in the dark.
(i) At the end of the incubation, wash the sections four times in TBS-T, add about 200 mL of secondary antibody labelled with horseradish peroxidase, and incubate the slides at room temperature for 30 min.
(j) Wash the enhanced sections five times in fresh TBS-T and stain the slides with hematoxylin compound and incubate it for 8–10 min.
(k) Wash the slides in fresh TBS-T and stain with eosin compound and incubate in for 3–5 min.
(l) Wash the slides again in fresh TBS-T and rehydrate by incubating in 95% ethanol for 2 min followed by 1 dip in 95% ethanol and 2 dips in 100% ethanol.
(m) Incubate the ethanol dehydrated sections in 40 mL of xylene in a glass jar for five minutes and allow the slides to air dry.
(n) Observe the staining pattern under the microscope.
